# The Pathophysiological Significance of Dual Inhibition of IL-17A and IL-17F in Psoriatic Arthritis and Axial Spondyloarthritis

**DOI:** 10.31138/mjr.020925.aar

**Published:** 2026-01-08

**Authors:** Olga Katsouli, Vasileios Lainis, Andreas V. Goules, Panayiotis G. Vlachoyiannopoulos

**Affiliations:** Department of Pathophysiology, Medical School, National and Kapodistrian University of Athens, Athens, Greece

**Keywords:** spondyloarthritis, psoriatic arthritis, interleukin-17A, interleukin-17F, dual inhibition, bimekizumab

## Abstract

**Objective:**

Spondylarthritides (SpA), including psoriatic arthritis (PsA) and axial spondyloarthritis (axSpA) are immune-mediated diseases in which the IL-23/IL-17 axis plays a central role. IL-17A inhibition is already a well-established and effective treatment strategy. Emerging evidence suggests that IL-17F, a cytokine closely related to IL-17A but less potent, is not a passive bystander but a key participant in chronic sustained inflammation and therapeutic escape, offering a compelling concept for dual inhibition. This review aims to examine the pathophysiological significance of dual inhibition of IL-17A and IL-17F in psoriatic arthritis and axial spondyloarthritis.

**Methods:**

Conducting a search through PubMed/MEDLINE, Scopus, and Web of Science, using keywords and Medical Subject Headings (MeSH) terms such as “spondyloarthritis”, “psoriatic arthritis”, “IL-17A”, “IL-17F”, “IL-23”, “IL-23 independent mechanisms”, “dual inhibition”, “pathogenesis”, “gut-joint axis”, “secukinumab”, “ixekizumab”, “bimekizumab”, “sonelokimab”, we select the relevant literature for this comprehensive pathophysiological narrative.

**Results:**

This review examines the distinct and synergistic functions of IL-17A and IL-17F in key tissues, including the synovium, enthesis, skin, gut, and eye, and their contribution to the paradoxical imbalance of bone remodelling. The advent of dual IL-17A/F inhibitors, particularly bimekizumab has revolutionised our therapeutic approach, offering a promising option for patients with difficult-to-treat SpA. We also discuss emerging technology agents, such as sonelokimab and izokibep.

**Conclusion:**

Inhibiting both IL-17A and IL-17F signifies not only a significant therapeutic advancement but also a vital strategy towards overcoming the limitations of our current armamentarium in achieving deeper and more sustained remission in SpA. Future studies and long-term data will be essential in determining the position of dual IL-17A/F inhibitors within treatment guidelines.

## INTRODUCTION

Spondyloarthritides encompass a group of related yet phenotypically distinct inflammatory disorders, including psoriatic arthritis (PsA), non-radiographic axial spondyloarthritis (nr-axSpA), radiographic axSpA (ankylosing spondylitis or AS), arthritis linked with inflammatory bowel disease (IBD), reactive arthritis, juvenile idiopathic arthritis, and acute anterior uveitis. These diseases exhibit shared immunological and inflammatory characteristics while presenting similar clinical features.^1^

Robust genetic evidence, along with preclinical and clinical studies, highlights the pivotal roles of interleukin IL-23 and IL-17 in the pathogenesis of SpA. This knowledge has facilitated the development of multiple effective therapies targeting IL-17A (secukinumab, ixekizumab), the IL-17 receptor A (brodalumab), and IL-23 (guselkumab, tildrakizumab, and risankizumab), resulting in significant changes to the therapeutic armamentarium for SpA.^2^

Nevertheless, real-world data show that a subset of patients continues to lack response to IL-17A blockade, due to alternative cytokine drivers or escaping pathways, or, less frequently,^3^ they experience a secondary loss of efficacy, attributed to the inherent plasticity of the underlying immunologic mechanisms, inflammation-independent structural progression, and, rarely, the presence of anti-drug antibodies.^4,5^ Therefore, new therapies will be needed to achieve better disease outcomes. Dual and bispecific IL-17A and IL-17F inhibitors, targeting IL-17A and IL-17F simultaneously, have been developed as promising and effective therapeutic weapons for improved disease management.^6^

In this review, we aim to emphasise the roles of IL-17A and IL-17F in PsA and axSpA, while presenting the rationale for their dual inhibition as a new strategy to address the treatment challenges faced in these diseases.

## RESEARCH STRATEGY

We conducted the literature search for this narrative review through PubMed/MEDLINE, Scopus, and Web of Science, using a combination of keywords and Medical Subject Headings terms (MeSH) such as “spondyloarthritis”, “psoriatic arthritis”, “IL-17A”, “IL-17F”, “IL-23”, “IL-23 independent mechanisms”, “dual inhibition”, “pathogenesis”, “gut-joint axis”, “secukinumab”, “ixekizumab”, “bimekizumab”, “sonelokimab” in March 2025, and updated in June 2025. Our search focused on randomised controlled trials, original articles, and high-impact narrative and systematic reviews published in English from September 2001 to June 2025, with some older references included for historical background. We also screened the reference lists of these articles to identify other relevant studies. To enhance coverage of open-access literature, an additional search was conducted in the Directory of Open Access Journals (DOAJ) in June 2025, using the same strategy. Finally, we carefully choose the selected literature to build a thorough pathophysiological narrative and include the most recent evidence for therapeutic strategies.

## IL-17 CYTOKINE FAMILY

### Overview and Evolutionary Diversification

**Figure 1.**
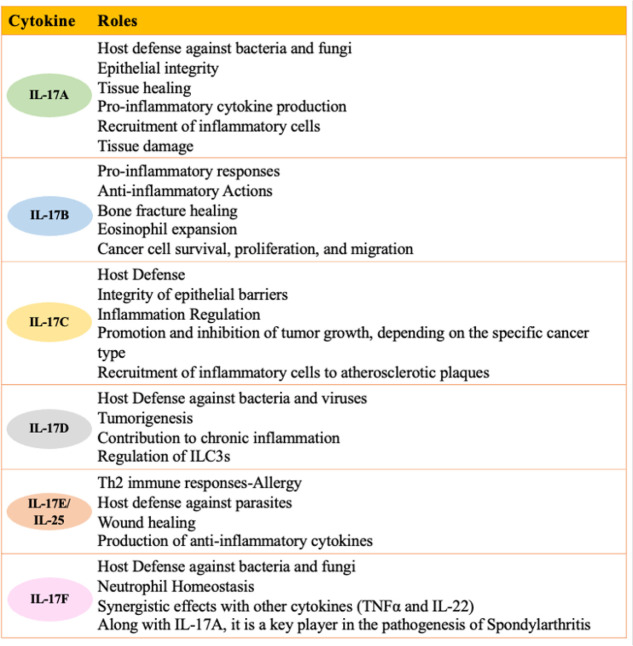
IL-17 family members and their roles.

The IL-17 cytokine family comprises six members— IL-17A through IL-17F—each with distinct yet often overlapping roles in immune regulation. IL-17A is the most biologically active cytokine in this family. IL-17F exhibits the highest structural similarity to IL-17A, with approximately 50% homology, since their respective genes are situated closely on the chromosome 6. These cytokines are produced by cells of the innate and adaptive immune system and are crucial for combating extracellular bacteria and fungi, particularly at barrier sites in the skin, gut, and lungs. In contrast, IL-17C and IL-17E (also known as IL-25), which are primarily secreted by epithelial and other non-hematopoietic cells, play a role in modulating immune responses to allergens and helminths through Th2 responses (**Figure 1**).^7–9^

The diversification of this cytokine family throughout the evolution of species likely represents a strategy to strengthen mucosal defence mechanisms, ensure redundancy, and facilitate tissue-specific modulation of inflammation. This reflects an evolutionary adaptation to specific microbial threats and homeostasis, balancing protection and pathology, such as preventing infection without triggering autoimmunity.

### IL-17 downstream

**Figure 2.**
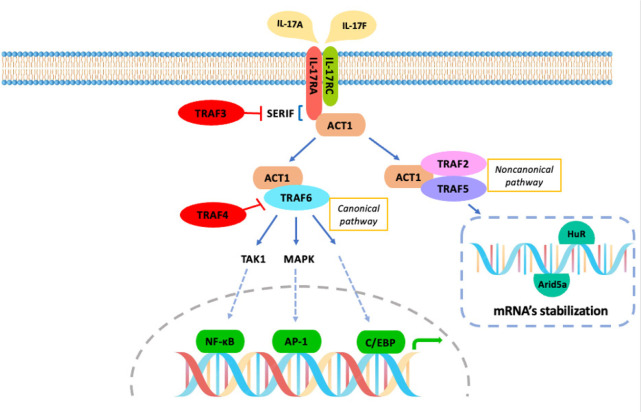
IL-17A/F signalling pathway.

IL-17A and IL-17F exist as disulfide-linked homodimers or as heterodimers of IL-17A/-17F. All variants of IL-17A and IL-17F cytokines interact through a receptor complex composed of IL-17RA and IL-17RC. IL-17RA is widely expressed, whereas IL-17RC expression is more tissue-specific, particularly in epithelial and mesenchymal cells of skin and synovium and to a lesser extent to entheseal fibroblasts and conjunctival, corneal, and retinal pigment epithelial cells or uveal stromal cells, aligning with the clinical manifestations of IL-17-mediated diseases.^10^ Both IL-17 RA and RC could be also expressed by innate immunity cells, like macrophages, monocytes and dendritic cells.^11^ IL-17F is thought to function similarly to IL-17A but with less potency, as it exhibits a lower affinity for the IL-17RA/RC complex.^12,13^ Upon binding, the IL-17R undergoes a conformational change that enables the recruitment of Act1, a key adaptor protein in signalling. Act1 attaches to IL-17RA through SEFIR (similar expression to fibroblast growth factor genes and IL-17R) domains and then recruits TNF receptor-associated factor 6 (TRAF6).^14,15^ This cascade activates the canonical NF-κB pathway, resulting in the transcription of pro-inflammatory cytokines (IL-6, GM-CSF), chemokines (CXCL1, CXCL2, CXCL8 (IL-8), and anti-microbial peptides (β-defensins, S100 proteins). The Act1-TRAF6 complex also engages MAP kinase signalling pathways, modulating gene expression and amplifying the combined effects of IL-17A/F with TNF-α and IL-1β.^16,17^ Moreover, IL-17 signalling enhances the expression of C/EBP transcription factors, essential for granulopoiesis and neutrophil survival, contributing to prolonged inflammatory responses.^18^ Finally, Act1 interacts with the TRAF2/TRAF5 complex, modulating the noncanonical pathway. Here, several RNA-binding proteins (HuR and Arid5a) play a role in stabilising mRNA. This characteristic of IL-17 signalling extends the half-life of short-lived inflammatory cytokines, clarifying why IL-17 signalling serves to amplify inflammation rather than initiate it (**Figure 2**).^19^

### Sources of IL-17A and IL-17F

IL-23 is a critical cytokine for the stabilisation, survival, and pathogenicity of specific IL-17-producing cells.^20^ Th17 cells are the primary source of IL-17A and IL-17F, and also secrete other cytokines such as IL-21, IL-22, IL-24, and GM-CSF. When IL-23 binds to its receptors on the cell membrane, it initiates a signalling cascade that activates the ROR-gamma t transcription factor, which is the master regulator of Th17 differentiation.^21,22^

It has been established that TGF-β and IL-6 promote the differentiation of non-pathogenic Th17 cells, while IL-23 drives them toward a pathogenic profile. The TGF-β/IL-23 ratio acts as a rheostat for the functional state of Th17 cells. An increase in this ratio leads to the formation of non-pathogenic Th17 cells, producing anti-inflammatory cytokines like IL-10, IL-21 and IL-17A, maintaining the mucosal integrity (such as in the gut) and tissue repairing and homeostasis. In the same time, a lower ratio shifts them to a pathogenic state, characterised by production of pro-inflammatory cytokines, such as GM-CSF, IL-22 and IFN-γ, promoting recruitment of inflammatory cells and contributing to autoimmunity and tissue damage.^23–25^

Similar to Th17 cells, Tc17 cells, a specific, less cytotoxic subset of CD8^+^ T cells, are notably responsive to IL-23 stimulation.^26^ They express RORγt and produce IL-17A, IL-17F, and IL-22, playing a role in chronic tissue inflammation.^27–29^

Innate lymphoid cells (ILCs) and innate-like lymphocytes (ILLs), including ILC3, gamma delta (γδ) T cells, mucosal-associated invariant T (MAIT) cells, and natural killer T (NKT) cells, represent additional sources of IL-17A and IL-17F.^30,31^ Recent studies indicate that these cells secrete IL-17A and IL-17F when stimulated by various cytokines, such as IL-7, IL-9, IL-12, IL-1β, and IL-18, rather than the typical IL-23. Consequently, while these cells may express the cell surface marker IL-23R, they can follow an alternative IL-23-independent pathway. This may explain the limitations of blocking IL-23 in managing patients with axSpA.^32^

## ROLE OF IL-17A AND IL-17F IN PSORIATIC ARTHRITIS AND AXSPA

### Overview of SpA pathogenesis

The central concept of SpA pathogenesis involves a multifactorial interplay between genetic predisposition (eg, HLA-B27), innate and adaptive immune activation (IL-23/IL-17 axis, innate-like T cells, mesenchymal cells), biomechanical stress at entheses, aberrant bone remodelling, and gut dysbiosis. In 1971, John Ball first proposed the enthesis as the primary target of inflammation in SpA.^33^ Later, in the early 2000s, Dennis McGonagle and colleagues expanded this idea, providing clinical, radiological, and histological evidence that enthesitis is central for the initiation and perpetuation of inflammation and damage in SpA pathogenesis.^34^ However, this enthesis-derived disease model cannot fully explain the sacroiliac joint inflammation, which is the main manifestation of axial SpA.^35^

During the last 20 years, current evidence suggests that gut inflammation plays an essential role in the disease pathogenesis. Nowadays, the “gut-joint” axis is broadly accepted and links the gut dysbiosis, subclinical inflammation of the gut, and synovitis.^36^ Impairment of the intestinal barrier structure and function leads to the translocation of microbial pathogen-associated molecular patterns (PAMPs) into the lamina propria. Altered microbiome promotes immunological changes in the gut.^37^ Activated Paneth cells produce IL-23 and IL-17. IL-23 causes differentiation of Th17, γδ T, and MAIT cells, amplifying IL-17 responses.^38,39^ Also, activated macrophages induce TNF-α production. Notably, IL-23 independent mechanisms also contribute to disease pathogenesis; innate-like lymphocytes, neutrophils, and mast cells can also produce IL-17 directly, bypassing IL-23 signalling, explaining residual disease activity in axSpA patients under IL-23 inhibitors.^32^

Activated cytokines, Th17 cells, and innate-like T cells can migrate and transport inflammation to other extraintestinal sites, such as axial/peripheral joints, eyes, or skin, inducing immune responses away from the gut.^37^ Furthermore, the similarities between normal human entheseal immune cells and those residing in the intestine raise the hypothesis of an additional “gut-enthesis” axis in SpA development, linking mucosal immunity and peripheral tissue inflammation.^38,39^

Beyond inflammation, cytokine-driven bone remodelling is central to disease pathogenesis and outcomes, reflecting a paradoxical process of simultaneous bone erosion and bone formation-ankylosis that characterises SpA. The dual role of IL-17A in promoting both processes is characteristic. IL-17A is implicated in both osteolysis, promoting osteoclastogenesis, and osteo-proliferation, stimulating the differentiation of osteoblastic mesenchymal cells, contributing to the distinct structural damage in SpA.^40^ IL-22 has been shown to promote osteoblastic differentiation and proliferation in mesenchymal stem cells and osteoblastic precursors, finally leading to osteoproliferation, as supported by in vitro^41^ and animal experimental findings.42 In vivo evidence remains limited and indirect, as indicated by the increase in IL-22-producing cells in the gut and peripheral blood of SpA patients, without a clear correlation with radiographic disease progression.^43,44^

### Evidence from animal models and human studies

Evidence from animal models has highlighted the importance of the IL-23/IL-17 axis in the pathogenesis of PsA and axSpA (**Table 1**).

**Table 1.** Summary of the known animal models highlighting the importance of the IL-23/IL-17 axis in the pathogenesis of PsA and axSpA.

**Animal Model**	**Genetic Feature**	**Key Pathological Findings**	**Role of IL-23/IL-17 Axis**	**Effect of IL-17/IL-23 Inhibition**
**HLA-B27 Transgenic Rats^45–49^**	Overexpression of HLA-B27 and β2-microglobulin	Colitis, arthritis, enthesitis, psoriasis, new bone formation	IL-23/IL-17 axis drives inflammation and bone formation	IL-17A inhibition: ↓ inflammation, damage, bone formation; delayed disease onset IL-23R blockade: effective only before disease onset (not after immunisation)
**SKG Mice (ZAP-70^w163c mutation)^50–52^**	Mutation affecting T cell selection, triggered by fungal/viral exposure	Enthesitis, arthritis, spondylitis, ileitis, no axial bone formation	Fungal component activates IL-23 → ↑IL-17, IL-22, mucosal dysregulation	IL-17A inhibition: reduces arthritis severity; highlights gut-joint axis and microbiota role
**IL-23 Minicircle Mouse Model^53^**	Overexpression of IL-23	Arthritis, enthesitis, spondylitis	Overproduction of IL-17A & IL-17F by γδ T cells and innate-like lymphocytes	IL-17 inhibition suppresses disease Single IL-17A inhibition: does not prevent IL-17F-driven bone formation
**Collagen-Induced Arthritis (CIA) Model^54–55^**	Autoimmunity via type II collagen	RA-like Inflammatory arthritis	Elevated IL-17 in serum and joints	IL-17A inhibition: partially reduces arthritis severity
**STAT3 Overexpression Model^56–59^**	Overexpression of STAT3 → hyperactive CD4^+^ T cells	Psoriasis, enthesitis, tendonitis, erosive arthritis	STAT3 promotes Th17 differentiation → ↑IL-17, IL-22, osteoclastogenesis	STAT3 Overexpression: ↑ inflammation Knockout: protection from arthritis and experimental autoimmune encephalomyelitis

HLA-B27: Human Leukocyte Antigen B27, IL-17A: Interleukin-17A, IL-23R: Interleukin-23 Receptor, IL-22: Interleukin-22, IL-17F: Interleukin-17F, STAT3: Signal Transducer and Activator of Transcription 3, Th17: T helper 17.

HLA-B27 transgenic rats characterised by overexpression of HLA-B27 b2 microglobulin developed colitis, arthritis, enthesitis, and psoriasis. Signs of peripheral and axial new bone formation were observed.^45,46^ Van Tok et al. suggested that prophylactic inhibition of IL-17A reduces joint inflammation, structural damage and periosteal new bone formation and delays the development of spondylitis and arthritis.^47^ Immunisation of these rats with heat-inactivated Mycobacterium tuberculosis led to the development of spondylitis and arthritis after 2–3 weeks, which could not be resolved after IL-23R blockade. In contrast, blocking IL-23R after immunisation but before the onset of clinical manifestations completely prevented the development of arthritis and spondylitis, highlighting the importance of IL-23 early in the disease course.^48,49^

SKG mice with ZAP-70w163c mutation, which affects T cells positive and negative selection, develop enthesitis, arthritis, spondylitis, and ileitis with no apparent signs of axial bone formation after exposure to fungi and viruses.^50^ Injection with curdlan, a component of the fungal wall, leads to the activation of the IL-23 axis, resulting in IL-17 and IL-22 overexpression and mucosal dysregulation.^51,52^ These animal models highlighted the role of IL-17 in arthritis after exposure to fungal cell wall components, the significance of inhibition of IL-17A in reducing the severity of arthritis, and the importance of the gut-joint axis since colonisation with specific microbiota, leading to the upregulation of IL-17 and the development of arthritis.^50–52^

The IL-23 minicircle mouse model, characterised by the overexpression of IL-23, was associated with developing arthritis, enthesitis, and spondylitis. The model highlights the production of IL-17A and IL-17F from γδ Τ cells and innate-like lymphocytes and the role of IL-17 inhibition in suppressing the disease course. However, inhibition of IL-17A did not prevent bone formation, which is considered IL-17F-driven.^53^

The Collagen–Induced Arthritis model, a key animal model for the pathogenesis of rheumatoid arthritis, has been used to study the role of IL-17 in inflammation. In this model, IL-17 levels in serum and joints are elevated, and the inhibition of IL-17A leads to a partial response to the severity of arthritis.^54,55^

STAT3 plays a key role in differentiating naive CD4 T cells into Th17.^56^ STAT3 animal models characterised by the overexpression of STAT3 show SpA features with psoriasis, enthesitis, and erosive arthritis. STAT3 overexpression models develop hyperactive CD4 cells that promote Achilles tendonitis through IL-17 and IL-22-dependent osteoclastogenesis and joint destruction.^57,58^ On the contrary, STAT3 knockout models exhibit protection from inflammatory diseases, including experimental autoimmune encephalomyelitis and arthritis.^59^

The above animal models propose that both IL-17A and IL-17F are key players in the pathogenesis of ax-SpA and PsA, with IL-17A being more important in the acute inflammatory phase. In contrast, the role of IL-17F emerges later during the disease course.^60^

### IL-17A and IL-17F in human skin and synovium

Both IL-17A and IL-17F have been detected in psoriatic skin lesions and synovial tissue. Although IL-17F is less potent than IL-17A, both induce a proinflammatory response with elevation of key inflammatory cytokines such as IL-6 and IL-8. The inflammatory capacity of IL-17F is 100 times weaker than that of IL-17A, whereas the concentration of IL-17F in tissues can be up to 30 times greater than that of IL-17A.^61^

The role of IL-17F in chronic inflammation was examined after inhibition of IL-17A and/or IL-17F in synoviocytes of PsA patients. Single inhibition of IL-17A significantly reduced the levels of inflammatory mediators such as IL-8, whereas single IL-17F inhibition had no significant effect. Contrariwise, dual inhibition of IL-17A and IL-17F resulted in greater downregulation of IL-6, IL-8, and MMPs.^62^ This underlines that both IL-17A and IL-17F contribute to chronic tissue inflammation synergistically. Additionally, dual inhibition shows a more potent anti-inflammatory effect on multiple inflammatory-related genes and causes a more significant reduction in neutrophil migration than the individual inhibition of each cytokine separately.^62^

Expression of IL-17A and IL-17F has been detected in psoriatic skin lesions, synovial tissue, and synovial fluid. The expression of IL-17F is greater than IL-17A in skin, whereas the levels of IL-17A are greater in the joint compartment, synovial tissue, and fluid.^63^ Chen et al. demonstrated that biopsies of skin and synovium collected from PsA patients with active PsO exhibited a higher IL-17F to IL-17A ratio in the skin. On the contrary, levels of IL-17A are more than 30-fold higher than IL-17F in the joint compartment.^64^ The above indicates that the role of IL-17F in chronic inflammation is more prominent in the skin than in the joint. Moreover, there is evidence from skin biopsies suggesting that IL-17A and IL-17F transcripts correlate with disease activity in psoriasis.^65^

### IL-17A and IL-17F in Enthesitis

Enthesis is a highly mechanically-stressed site vulnerable to microinjury, where the IL-23/17 axis may be adjusted to support its homeostasis. Enthesitis is the cardinal pathological process in SpA, triggered predominantly by an innate immune response. PGE2 and IL-23 are essential early mediators, activating resident immune cells to produce IL-17A and other cytokines.^66^ In their mice arthritis experiments, Cambré et al. suggested that mesenchymal stromal cells detect mechanical strain in mechano-sensitive areas, such as entheses, and transform it into biochemical signals, like chemokines, which trigger local inflammation. Prolonged inflammation at the entheseal sites results in new bone formation and, to a significantly lesser degree, bone erosions.^67^

New bone formation and bone erosion can occur simultaneously, with IL-17A playing a dual role in promoting both processes. Recent research has identified enthesis-resident ILC3 and γδ T cells in humans that produce IL-17A. IL-17A likely enhances enthesitis by promoting the release of various cytokines from local mesenchymal cells either boosting the differentiation and activation of osteoblasts, which is associated with osteogenesis and bone formation, or stimulating the expression of receptor activator of NF-κΒ (RANKL), promoting osteoclastogenesis and subsequent bone erosions in these areas.^40,66,68,69^ Nevertheless, our comprehension of IL-17F biology at the human enthesis remains limited. McDermott et al. conducted the initial functional study on the immunobiology of IL-17F in the human enthesis. Their research revealed that the normal enthesis contains both innate and adaptive T-cells, primarily CD4 and γδ T-cells, which can trigger the expression of IL-17F and IL-17A upon stimulation with IL-23.^70^ However, no evidence of IL-23-induced production of IL-17F was found. Consistent with the experiments of Bridgewood et al., IL-17A or IL-17F dramatically synergise with TNF to induce CCL20 from enthesis stromal cells, a chemokine that mediates the further migration of IL-17-producing lymphocytes to entheseal tissues. No further effects were observed with the combination of IL-17A and IL-17F.^71^

### IL-17A and IL-17F in Gut Inflammation

Gut inflammation results in the loss of mucosal barrier, allowing dietary antigens and microbes to enter the bloodstream, leading to immune system dysregulation.^72–74^ The role of IL-17 in the gut is uncertain, as it exerts both pro-inflammatory and protective characteristics. The IL-23 independent production of IL-17A by the innate-like immune cells maintains the integrity of the mucosal barrier, exhibiting anti-inflammatory and homeostatic properties.^75^ On the contrary, IL-23-dependent production of IL-17A by Th17 cells leads to gut inflammation.^76,77^ These could explain why anti-IL17A treatment with secukinumab worsens Crohn’s disease, while inhibition of IL-23 is an IBD treatment.^78,79^

It is well established that IL-17A exerts a protective role for mucosal barrier integrity, by regulating the expression of the tight junction proteins occludin and claudin, maintaining in this way barrier integrity.^80^ In addition, IL-17A and IL-22 induce the production of protective antimicrobial mediators like defensins, mucus, and IgA, inducing the recruitment of neutrophils at sites of damage and infection.^80^ The above highlights the protective role of IL-17A in wound healing in active colitis and justifies the avoidance of IL-17A inhibitors in these patients.^81^

Similar to IL-17A, IL-22 maintains mucosal homeostasis by inducing the production of mucus and antimicrobial mediators, preserving mucosal barrier integrity, and facilitating pathogen clearance. It promotes the production of hemopexin, a protein that restricts iron availability for bacterial overgrowth and downregulates the C3 complement gene.^82^ Moreover, IL-22 affects T helper cell polarisation, diminishing Th1 responses while promoting their regulatory phenotype. T-cell-mediated colitis in animal models indicates that inhibiting IL-17A results in decreased IL-22 levels, upregulating Th1 pathways, which leads to gut inflammation and reduced epithelial regeneration.^83^

Our understanding of the role of IL-17F in colonic inflammation remains unclear. Seiderer et al. reported that the expression of intestinal IL-17F mRNA was significantly elevated by 4.4 times in inflamed colonic lesions compared to uninflamed biopsies in CD, but not in Ulcerative Colitis (UC).^84^ Nevertheless, one report found that the IL-17F His161Arg polymorphism was not linked to IBD susceptibility.^84^ Conversely, Arisawa et al. offered the first evidence that polymorphisms in IL-17A (rs2275913, G-197A) and IL-17F (rs763780, 7488T/C) genes are independent risk factors for UC development and have significant associations with the pancolitis phenotype.^85^

The differing effects of IL-17 and IL-17F have also been observed in acute experimental dextran sulphate sodium (DSS)-induced colitis, where IL-17F appears to act as a pathogenic factor. IL-17F-knockout mice showed protection with reduced expression of CCL2, CCL5, and CCL7, while IL-17 deficiency resulted in increased colon damage.^86^ These results suggest that IL-17 is protective, whereas IL-17F may exacerbate intestinal inflammation. However, the evidence so far remains inconclusive.

### IL-17A and IL-17F in Uveitis

Th17 cells and their related cytokines are important inflammatory mediators in the pathogenesis of autoimmune uveitis, initiating and perpetuating ocular inflammation. IL-17 induces the production of inflammatory cytokines from astrocytes and retinal pigment epithelial cells and negatively affects retinal ganglion cells, leading to visual impairment.^87–89^

Elevated levels of serum IL-17A and IL-17F, found in both blood and intraocular spaces, have been identified in patients with active uveitis, making them important targets for treatment.^90–92^

Experimental autoimmune uveitis animal models highlight the pathogenetic role of IL-23-dependent Th17 cells, while IL-17 inhibition reduces ocular inflammation in mice.^93^ Moreover, STAT3-knockout mice, a key transcription factor for Th17 differentiation, do not develop uveitis.^94^

Beyond its pro-inflammatory role, IL-17 exhibits immunoregulatory properties. IL-17A stimulates the synthesis of IL-24, creating a negative feedback mechanism that suppresses the production of inflammatory cytokines such as IL-17A, IL-17F, and GM-CSF.^95^ Additionally, IL-17 promotes the production of Foxp3+ T regulatory cells.^96^ This illustrates the dual role of IL-17 in the pathogenesis of uveitis, partially explaining the variable results of inhibiting IL-17A with Secukinumab and Ixekizumab in uveitis.

### Contribution to bone erosion, new bone formation, and inflammation

IL-17A promotes osteoclastogenesis. IL-17A induces the expression of receptor activator of NF-κB ligand (RANKL) on osteoblasts, resulting in the production of mature osteoclasts from osteoclastic precursors.^97–99^ Moreover, IL-17 promotes, via chronic inflammation, the matrix turnover and activates MMPs, leading to cartilage loss. IL-17A promotes the inhibition of Wnt signalling, leading to the inhibition of osteoblasts.^98,100^ Therefore, inhibition of IL-17A results in the reduction of radiographic progression. According to the PSARTROS study, after 24 weeks of treatment with secukinumab in patients with PsA, there was no progression in anabolic and catabolic bone changes in the joints.^101^ The role of IL-17A in bone formation is not fully understood, as there are many contradictory experimental results. IL-17A promotes the activation and differentiation of osteoblasts. IL-17 induces the osteoblastic differentiation of the mesenchymal stem cells, in collaboration with other cytokines like TNF-α.^102^ In addition, IL-17A promotes osteogenesis via the activation of osteoblasts through the JAK2/STAT3 pathway.^103^

IL-17F is considered a weaker mediator of osteogenesis and osteoclastogenesis. However, Shah et al., recently described that both IL-17A and IL-17F have an equal effect on progenitor cells from the periosteum. Th17 and γδ Τ cells supernatants and serum from patients with AS stimulated in vitro bone formation. Double inhibition of IL-17A and IL-17F suppressed bone formation more potently than single inhibition alone.^104^ Besides the role of IL-17A in bone remodelling, bone formation in the entheses also depends on another cytokine, IL-22. Entheseal-resident immune cells produce IL-22, promoting the osteogenic differentiation of mesenchymal cells and their migration to sites of bone repair, contributing to inflammatory osteogenesis and aberrant bone formation in SpA.^105–107^

**Table 2** summarises the known properties of IL-17A and IL-17F in the skin, synovium, entheses, eye and gut.

**Table 2.** Summary of the known properties of IL-17A and IL-17F in human skin, synovium, entheses, eye, and gut.

**Tissue/Organ**	**IL-17A properties**	**IL-17F properties**
**Skin^61,63–65^**	Central role in psoriasis Promotion of keratinocyte proliferation, production of inflammatory cytokines (e.g., IL-6, TNF-α) and chemokines Contribution to chronic inflammation and hyperkeratosis	Overlapping functions with IL-17A but less potent Higher expression of IL-17F than IL-17A in psoriatic lesions (up to ~30× IL-17A) Prominent role in chronic inflammation in skin
**Synovium^62,64^**	Powerful driver of synovial inflammation and tissue remodelling	Lower expression than IL-17A in joint compartment, synovial tissue, and fluid Synergistic effect with IL-17A
**Entheses^40,66–71^**	Recruitment of immune cells to the entheses Stimulation of osteoclastogenesis and bone erosion, parallel with osteogenic differentiation of mesenchymal cells, inflammatory osteogenesis and aberrant bone formation in SpA	Weaker mediator of osteogenesis and osteoclastogenesis Acts synergistically with IL-17A and TNF promoting inflammatory responses at the entheses Recently description of its equal effect with IL-17A on progenitor cells from the periosteum
**Eye^87–96^**	Implication in ocular inflammation (uveitis) Induction of the production of inflammatory cytokines from retinal pigment epithelial cells	Potentially contribution to ocular inflammation, similar with IL-17A
**Gut^72–86^**	Maintenance of intestinal microbiota homeostasis Maintenance of the epithelial barrier in the gut Contribution to IBD when dysregulated	Unclear role Possible similarities with IL-17A functions Possible exacerbation of intestinal inflammation, especially in CD

IL-6: Interleukin-6, TNF-a: Tumour Necrosis Factor-a, IL-17A: Interleukin-17A, IL-17F: Interleukin-17F, SpA: Spondyloarthritis, IBD: Inflammatory Bowel Disease, CD: Crohn’s Disease.

## THERAPEUTIC TARGETING OF IL-17 PATHWAYS

### Existing Biologics Targeting IL-17 Pathway

IL-17A has been identified as a driver of joint and skin inflammation in SpA, leading to the successful development of IL-17A inhibitors to treat these patients.

In early 2015, Secukinumab became the first IL-17A inhibitor approved by the FDA and EMA for treating patients with plaque PsO.^108^ By 2016, it also gained approval for axSpA and PsA treatments, showing efficacy across various disease activity measures, marked improvements in musculoskeletal areas (ACR20 response: 62.6%, 55.5% and 27.4%, ACR50 response: 39.6%, 35.9% and 8.1%, Enthesitis resolution: 55.7%, 54.6% and 35.4% and Dactylitis resolution: 65.9%, 57.5% and 32.3% in secukinumab 300mg, 150mg and placebo arms respectively at week 16 in FUTURE 5 trial^109^; ASAS20 response: 44% vs 21% and ASAS40 response: 26% vs 8% in secukinumab 150mg vs placebo arms respectively at week 16 in MEASURE 2 trial),^110^ and significant progress in psoriasis, with PASI100 scores achieving 40–50% and maintained for a minimum of 2 years of treatment.^111,112^ In the EXCEED trial that compared Secukinumab with Adalimumab monotherapy over 52 weeks for patients with PsA, Secukinumab did not show significant superiority to Adalimumab regarding musculoskeletal measures (ACR20 response: 76.4% vs 68.3% (p=0.175), ACR50 response: 28.2% vs 17.7%, (p=0.06). However, it demonstrated improved skin responses, as indicated by the PASI100 score (39.1% vs 23.8%, p=0.013).^113^ Regarding radiographic progression, the FUTURE 5 trial and its 2-year extension investigating the efficacy of Secukinumab in patients with PsA, using the mean change in van der Heijde-modified total Sharp score (vdH-mTSS), showed that all treatment arms provided sustained clinical efficacy and low radiographic progression through 52 weeks and 2-year follow-up.^114^ Comparing the spinal radiographic progression in axSpA patients treated with secukinumab vs adalimumab, the SURPASS IIIb study showed there was no significant difference between the treatment arms at week 104.^114^

Likewise, Ixekizumab, another IL-17A inhibitor, has shown remarkable effectiveness in treating axSpA and PsA. The COAST-V and COAST-W trials demonstrated significant efficacy in bDMARD-naïve and anti-TNF-experienced axSpA patients (ASAS40 response: 46.5% and 38.7%, respectively, at week 52).^116^ Studies on PsA indicated improvements in joint (ACR70 responses: 34–35% in biologic naïve vs 20–32% in TNFi experienced PsA at 52 weeks) and skin symptoms (PASI100 remission was achieved in 42.5% and 52.5% with ixekizumab Q4WK and Q2WK, respectively, and in 23.5% with adalimumab, versus 1.5% with placebo (p ≤ 0.001 for each ixekizumab arm, p < 0.01 for adalimumab). At 24 weeks, both ixekizumab arms (Q4WK and Q2WK) showed significant enthesitis improvement compared to placebo (42.6% and 38.6% respectively, vs 19.3%), while the active control arm treated with adalimumab achieved a 33.3% resolution of enthesitis, which was not significantly different from placebo. Enthesitis responses were maintained at 52 weeks (51.3% and 42.6%, respectively). Regarding dactylitis resolution: in both ixekizumab arms, 79.5% and 76.9%, versus placebo 25% (p < 0.001) at 24 weeks, and these improvements were maintained at 52 weeks (81.1% and 75%, respectively).^117^ A direct head-to-head trial indicated that ixekizumab outperformed adalimumab in PsA patients, leading to greater improvements in clinical outcomes and physical function (ACR50+PASI100: 36% vs 27,9% and DAPSA remission: 26,5% vs 18%, in ixekizumab vs adalimumab arms, respectively), suggesting it may serve as an ideal first-line monotherapy.^118^ Van der Heijde et al. evaluated the long-term effect of ixekizumab on spine radiographic progression in axSpA patients, using the modified Stoke Ankylosing Spondylitis Spinal Score (mSASSS). The vast majority of patients receiving ixekizumab showed no radiographic progression through 2 years of follow-up.^119^

Netakimab is another humanised monoclonal antibody targeting IL-17A. It has been approved for rx-axSpA, PsA, and psoriasis in Russia and Belarus following the successful completion of the ASTERA, PATERA, and PLANETA trials, respectively. However, it remains unapproved by the FDA and EMA.^120–122^

While the pure inhibition of IL-17A has revolutionised the management of SpA patients, especially those who have failed TNFi therapy, it does not influence signalling through other IL-17 family members. Brodalumab was the inaugural biologic to target additional IL-17 family members by focusing on the IL-17RA/IL-17C complex, effectively blocking the binding of the IL-17A, IL-17F, IL-17C, and IL-17E subunits.^123^ In 2017, the FDA and EMA approved it for treating moderate-to-severe psoriasis, accompanied by a black box warning regarding suicidal ideation and behavior.^124,125^ Its superiority over Ustekinumab has been confirmed in three phase III studies (AMAGINE-1, AMAGINE-2, and AMAGINE-3).^126,127^ Although it has not been as thoroughly studied as other IL-17 inhibitors, Brodalumab has demonstrated significant efficacy in both axSpA and PsA in phase III trials; it is, however, only approved for PsA in Japan.^128,129^

Overall, trials on the single inhibition of IL-17A have demonstrated strong efficacy in the skin, joints, dactylitis, and enthesitis, reducing the inflammatory burden of the disease and improving clinical and patient-reported outcomes. Concerning axial involvement, blocking IL-17A alone has shown high efficacy in reducing MRI-detected inflammation and consequently spinal pain, along with significant improvements in ASAS20 and ASAS40 responses, both in biologic-naïve and TNFi non-responders, with comparable effects to TNFi in halting the radiographic progression. The outcomes of single inhibition of IL-17A in the uveitis remain limited and inconsistent.^109–129^

### The First Dual Inhibitor of IL-17A and IL-17F: A New Kid on the Block

Recent therapeutic advances have led to the development of dual neutralising agents designed to target both IL-17A and IL-17F, to achieve a more extensive and robust suppression of IL-17-mediated inflammation, preventing secondary loss of therapeutic efficacy. Bimekizumab is the first humanised IgG1/κ monoclonal antibody that specifically and strongly binds to IL-17A, IL-17F, and IL-17AF, inhibiting their interaction with the receptor.^130^ Unlike the IL-17RA inhibitor Brodalumab, Bimekizumab spares IL-17E, known for its anti-inflammatory characteristics.^131^ Initially, it was approved for treating psoriasis and demonstrated its superiority over anti-TNF (PASI90: 86.2% for both groups of bimekizumab vs 47.2% for adalimumab, at week 16), anti-IL-23 (PASI90: 85% for bimekizumab vs 50% for ustekinumab at week 16), and various anti-IL-17 therapies (PASI100: 61.7% for bimekizumab vs 48.9% for secukinumab at week 16 and PASI100: 67% for bimekizumab and 46.2% for secukinumab at week 48) in multiple phase III clinical trials (BE SURE, BE VIVID, BE RADIANT, respectively).^132^

In June 2023, the European Commission (EC) approved marketing authorisation for Bimekizumab to treat adults with active PsA and axSpA, including nr-axSpA and r-axSpA.

In the BE OPTIMAL trial—a 52-week randomized, double-blind, placebo-controlled study with Adalimumab as an active reference—Bimekizumab demonstrated an ACR50 response in 44% of biologic-naïve PsA patients by Week 16, while over 60% reached PASI90. The ACR50 response rate for the Adalimumab group was comparable at 46%. Bimekizumab also provided rapid and significant improvement in other PsA domains, including enthesitis (enthesitis resolution: 50% vs 35% vs 50% for bimekizumab, placebo and adalimumab arms, respectively) and dactylitis (dactylitis resolution: 76% vs 51% vs 82% for bimekizumab, placebo, and adalimumab arms, respectively), by Week 16.^133^ Even in populations with PsA resistant to TNF inhibitors (as seen in the BE COMPLETE trial), Bimekizumab demonstrated strong clinical effectiveness, achieving a 43% ACR50 and 69% PASI90 response rate by Week 16.^134^

Regarding axSpA, dual inhibition using Bimekizumab resulted in significant improvements in ASAS responses, BASDAI, pain relief and quality of life compared to placebo in BE MOBILE 1 (nr-axSpA) and BE MOBILE 2 (r-axSpA) trials, regardless of any previous exposure to anti-TNF. ASAS40 response was sustained from Week 16 (nr-axSpA: 47.7%; r-axSpA: 44.8%) to Week 52 (nr-axSpA: 60.9%; r-axSpA: 58.4%). A significant proportion of patients achieved BASDAI50 at Week 52, in both treatment arms (nr-axSpA: Bimekizumab: 53.9%, Placebo/Bimekizumab: 49.2%; r-axSpA: Bimekizumab: 53.8%, Placebo/Bimekizumab: 62.2%). MRI also confirmed a rapid and significant reduction in inflammation of the spinal and sacroiliac joints.^135^ At week 16, patients with nr-axSpA and r-axSpA showed greater mean reductions from baseline in MRI Spondyloarthritis Research Consortium of Canada (SPARCC) sacroiliac joint (SIJ) inflammation scores with bimekizumab compared to placebo (nr-axSpA: –‍.3 vs –‍.5; r-axSpA: –5.6 vs +1.1, respectively). Reductions from baseline were also observed in mean MRI Berlin spine scores with bimekizumab vs placebo (nr-axSpA: –0.7 vs –‍.1; r-axSpA: –2.3 vs 0.0, respectively). These significant reductions observed at week 16 were maintained and improved at week 52 for both treatment groups (nr-ax-SpA: 79.5% for bimekizumab vs 56.3% for placebo/bimekizumab; r-ax-SpA: 75% for bimekizumab vs 66.7% for placebo/bimekizumab).^136^

Until recently, there had been no direct head-to-head studies of Bimekizumab in PsA and axSpA. On September 30, 2024, the global biopharmaceutical company marketing Bimekizumab announced the launch of the BE BOLD trial (NCT06624228), which is the first Phase IIIb head-to-head study designed to compare the efficacy and safety of Bimekizumab versus Risankizumab, an IL-23 inhibitor, in patients with active PsA. The completion of clinical trial and results are anticipated in 2026.^137^ **Table 3** summarises the clinical trials of Bimekizumab and Sonelokimab in PsO, PsA and axSpA.

**Table 3.** Clinical trials of Bimekizumab and Sonelokimab in PsO, PsA, and axSpA.

**Drug**	**Trial Name / Phase**	**Indication**	**Design & Comparator**	**Key Outcomes**	**Comments**
**Bimekizumab**	BE SURE (Phase III)^132^	PsO	Placebo, Etanercept	Superior PASI90/100 vs Etanercept; rapid and sustained skin clearance	Demonstrated superiority over anti-TNF therapy
	BE VIVID (Phase III)^132^	PsO	Placebo, Ustekinumab	Superior PASI90/100; sustained responses up to 48 weeks	High efficacy, favourable safety profile
	BE RADIANT (Phase III)^132^	PsO	Secukinumab	Superior skin clearance with Bimekizumab	Head-to-head with anti-IL-17A monoclonal antibody
	BE OPTIMAL (Phase III)^133^	PsA	Placebo, Adalimumab	ACR50: 44% Bimekizumab vs 46% Adalimumab; PASI90 >60%; rapid improvement in enthesitis, dactylitis	Biologic-naïve PsA patients
	BE COMPLETE (Phase III)^134^	PsA	Placebo	ACR50: 43%; PASI90: 69% at Week 16	TNF inhibitor-resistant PsA patients
	BE MOBILE 1&2 (Phase III)^135^	AxSpA	Placebo	Significant improvements in ASAS40, BASDAI, BASMI, pain, QoL; rapid MRI inflammation reduction	nr-axSpA and r-axSpA populations
	BE BOLD (Phase IIIb, ongoing)^137^	PsA	Risankizumab	Head-to-head comparison; results expected in 2026	First direct comparative trial vs IL-23 inhibitor
**Sonelokimab**	Phase IIb (Psoriasis)^145^	PsO	Secukinumab	PASI90: ~77%; PASI100: ~58% at Week 12	Comparable safety to secukinumab
	ARGO (Phase II)^146^	PsA	Placebo	ACR50: 60%, ACR70: ~40%, PASI90: >80%, PASI100: >60% at Week 24	Trispecific nanobody targeting IL-17A/F and IL-17F
	IZAR-1 & IZAR-2 (Phase III, ongoing)^147^	PsA	Placebo, Risankizumab (IZAR-2 active arm)	Primary endpoint ACR50; results expected in 2026	Biologic-naïve and anti-TNF inadequate responders
	S-OLARIS (Phase II, ongoing)^148^	AxSpA	Placebo	Evaluating ASAS40, BASDAI endpoints	Initial axSpA efficacy data pending

PsO: Psoriasis, PsA: Psoriatic Arthritis, AxSpA: Axial Spondyloarthritis, PASI: Psoriasis Area and Severity Index, anti-TNF: anti-Tumour Necrosis Factor, anti-IL-17A: anti-Interleukin-17A, ACR50: American College of Rheumatology 50% response criteria, nr-axSpA: non-radiographic axial spondyloarthritis, r-axSpA: radiographic axial spondyloarthritis, ASAS: Assessment of SpondyloArthritis International Society, BASDAI: Bath Ankylosing Spondylitis Disease Activity Index, BASMI: Bath Ankylosing Spondylitis Metrology Index, QoL: Quality of Life, MRI: Magnetic Resonance Imaging, IL-23: Interleukin-23, ACR70: American College of Rheumatology 70% response criteria.

### Safety of Dual Inhibition

Like other IL-17 pathway inhibitors, concerns exist regarding upper respiratory infections, candidiasis, and the potential onset of inflammatory bowel disease (IBD). Generally, the safety data from most clinical studies of Bimekizumab align closely with those of single IL-17A inhibitors. Among PsA patients, 14.6% reported upper respiratory tract infections, while the rate was 16.3% for patients with axSpA. Oral candidiasis occurred in 14.6% of PsA patients and 3.7% of those with axSpA, with no reported cases of invasive candid-iasis.^138–140^

It is widely recognised that IL-17 contributes to the repair of mucosal surfaces and intestinal mucosa integrity. Consequently, it paradoxically acts as a protective factor in the development of IBD. In light of these findings, anti-IL-17 agents have been linked to the onset of new or previously undiagnosed IBD. Nonetheless, patients with autoimmune diseases are at an increased risk for IBD due to overlapping susceptibility genes.^141^ In their first meta-analysis exploring the risk of new-onset IBD related to anti-IL-17 agents, which included 16,690 patients with existing autoimmune conditions, Yamada et al. reported an incidence rate of 2.4 cases per 1000 patient-years. This rate was low and not statistically significant compared to placebo.^141^ Furthermore, research indicates that dual inhibition of IL-17A and IL-17F does not seem to increase the risk of IBD compared to single IL-17A inhibition.^142^

Uveitis is the most common extra-musculoskeletal manifestation among patients with axSpA and a therapeutic challenge. Data from the BE MOBILE and BE AGILE trials suggest that the incidence of uveitis was significantly lower in patients treated with Bimekizumab compared to placebo, particularly in individuals without a history of uveitis, and was numerically lower in those with a prior history. The above suggests that dual inhibition of IL-17A and IL-17F with Bimekizumab possibly confers positive effects for uveitis in patients with axSpA.^143^

While research on tumorigenicity has yet to be performed, a few neoplasms, mainly basal cell carcinomas (0–1%), have been noted in the Bimekizumab treatment group.^135^

### Emerging Agents Targeting IL-17 Pathway

The evolution from single IL-17A to dual IL-17A/F or multi-functional agents signifies a pursuit of improved efficacy, increased durability, and superior patient outcomes while mitigating infection risks.

Nanobodies, small antibody fragments derived from a unique type of antibody found naturally in camelids, represent a novel class of targeted antibody-like therapeutics. Unlike traditional antibodies, nanobodies consist of a single heavy chain variable domain, making them ten times smaller than conventional antibodies. As a result, they are more effective at penetrating tissues such as joints, skin, and entheses, exhibiting greater stability, higher specificity, and lower immunogenicity. Research into the use of nanobodies for treating autoimmune diseases is ongoing.^144^ Sonelokimab (M1095/ALX-0761) is a trispecific nanobody that selectively binds to human IL-17F, human IL-17A and IL-17A/F, and human serum albumin for prolonged plasma half-life.^145^ The phase II ARGO trial (NCT05640245), including 207 patients with active PsA, indicated that Sonelokimab attained an ACR50 response in 60% of patients by Week 24. Over 80% and 60% of those treated reached PASI90 and PASI100, respectively. Around 40% of patients achieved the ACR70 outcome by Week 24.^146^ Following these results, the Phase 3 IZAR program announced in November 2024 is expected to recruit approximately 1,500 adult patients through two trials: IZAR-1 (NCT06641076) for biologic-naïve patients and IZAR-2 (NCT06641089) for inadequate responders to anti-TNF, using Risankizumab as an active reference arm. Both trials’ primary endpoint results (ACR50) are expected in 2026.^147^

An affibody is a small, engineered protein (approximately 6–7 kDa) that imitates the binding ability of an antibody to a specific target, though it is not a true antibody. It is constructed from a scaffold originating from a bacterial protein known as staphylococcal protein A, which is then modified to bind targets (such as IL-17A) with 1,000-fold higher affinity.^149^ Izokibep, a small protein that inhibits IL-17A, demonstrated promising efficacy in a double-blind, placebo-controlled, multicentre phase 2b/3 trial (NCT04713072). It met not only the primary endpoint of ACR50 but also achieved responses for the challenging ACR70, PASI90, and PASI100 endpoints. It was well tolerated, with a safety profile generally consistent with other IL-17A inhibitors.^150^

Recently, the phase II clinical trial of Izokibep in SpA (NCT04795141) was terminated by the sponsor, while its phase 2b/3 trial in non-infectious, non-anterior uveitis (NCT05384249) failed to meet the primary endpoint of statistically significant improvement compared to placebo at week 24.^151^

## PATHOPHYSIOLOGICAL INSIGHTS FROM DUAL INHIBITION

As mentioned above, IL-17A and IL-17F are key pro-inflammatory cytokines that signal through IL-17 receptor complexes, primarily composed of IL-17RA and IL-17RC subunits. Upon ligand binding, these receptors recruit Act1, which interacts with TRAF family members, especially TRAF6, initiating signalling cascades that lead to the transcription of genes for inflammatory mediators such as cytokines, chemokines, and metal-loproteinases.^107^ Notably, IL-17RA contains a unique C/EBPβ activation domain (CBAD) that can also bind TRAF3, a negative regulator competing with TRAF6. This interaction provides a regulatory checkpoint that can attenuate IL-17-driven signalling.^18^ However, blocking IL-17RA—while therapeutically beneficial—may inadvertently allow IL-17 to signal through IL-17RC homodimers or alternative receptor complexes such as IL-17RC combined with other co-receptors (eg, IL-17RD) or through non-canonical IL-17 signalling via adaptor proteins like Act1, indicating a potential escape mechanism that could undermine therapeutic efficacy.^10,15,152^

Both IL-17A and IL-17F are pro-inflammatory cytokines with modest activity. Still, their pro-inflammatory effect increases dramatically when they synergise with other inflammatory cytokines, such as IL-1, TNFα, and IL-6, according to data from animal models and human studies. IL-17A is a more potent inflammatory cytokine, but IL-17F levels are significantly higher in the serum and skin lesions of patients with PsO and PsA.^61^ Specifically, IL-17F mRNA levels are 2.7-fold higher in the skin than IL-17A, whereas IL-17A mRNA levels and protein levels are 17.3-fold higher in synovium and 37.4-fold higher in synovial fluid than IL-17F.^64^

Moreover, studies have shown that in immune cells, such as Th17, an autocrine regulatory feedback loop tunes their pathogenicity.^153^ After the binding of IL-17A to the IL17RA/IL17RC heterodimer, the activation of NF-κB leads to the secretion of IL-24. IL-24 acts in an autocrine way and suppresses the production of the key inflammatory cytokines IL-17F and GM-CSF from Th17 cells. Blocking IL-17A alone terminates this autocrine regulatory loop, allowing the expression of inflammatory cytokines such as IL-17F without the regulatory effect of IL-24.^95^ The above highlights that IL-17A alone may have limitations and proposes a pathophysiological explanation for the superiority of blocking both IL-17A and IL-17F.

IL-17A homodimers demonstrate the highest proinflammatory potential, followed by IL-17A/IL-17F heterodimers and IL-17F homodimers. Inhibiting both IL-17A and IL-17F in human Th17 supernatants results in greater suppression of proinflammatory chemokines and cytokines compared to inhibiting IL-17A or IL-17F alone.^154^

Burns et al.^155^ demonstrated for the first time that IL-17A^+^IL-17F^−^, IL-17A^+^IL-17F^+^, and IL-17F^+^IL-17A^−^ CD4^+^T cells display different cytokine profiles. IL-17F^+^CD4^+^T cell populations displayed lower frequencies of IL-10 and GM-CSF and higher frequencies of IFN-γ compared to IL-17A^+^IL-17F^−^CD4^+^T cells. Consistent with previous research, they demonstrated that IL-17F stimulates synovial fibroblasts to secrete IL-6 and IL-8, a process that is amplified in the presence of TNF-α. Their experiments showed that blocking IL-17F alone does not influence cytokine secretion; however, the combined blockade of IL-17A and IL-17F using Bimekizumab significantly reduces the secretion of IL-6, IL-8, CXCL1, CXCL5, and CCL20 by fibroblasts more effectively than blocking IL-17A alone.^155^

**Figure 3.**
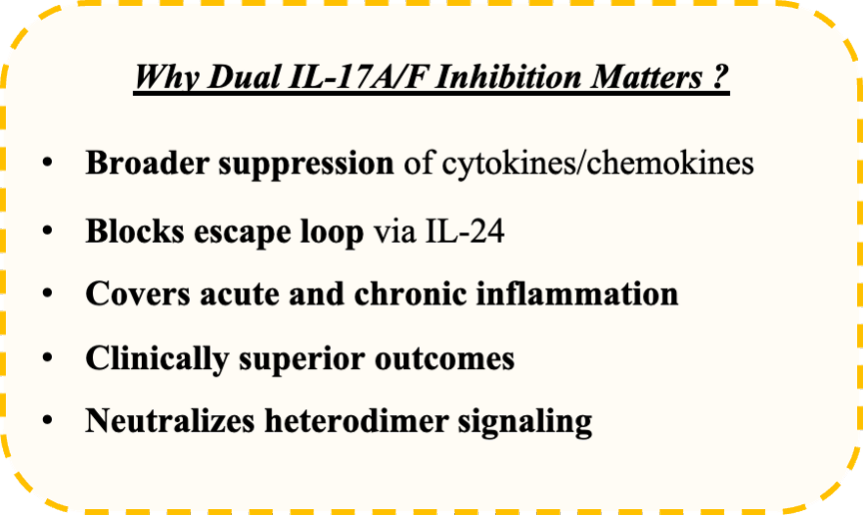
Why Dual IL-17A/F Inhibition Matters?

Interestingly, IL-17 production varies over time. It is believed that IL-17A is produced rapidly in response to the stimulation of Th17 cells. In contrast, the production of IL-17F gradually increases, reaching its peak levels later in the disease course. Additionally, upon continued stimulation, T cells produce IL-17F rather than IL-17A. Thus, IL-17A may play a critical role in acute inflammation during the early stages of the disease, while IL-17F becomes more significant in the later stages and during chronic inflammation.^26^ Therefore, targeting IL-17A and IL-17F with Bimekizumab and Sonelokimab could benefit patients with chronic and refractory disease (**Figure 3**).

## CONCLUSION

The therapeutic targeting of IL-17 pathways has revolutionised the management of spondyloarthritis and related inflammatory diseases, marking a significant advancement beyond traditional anti-TNF therapies, controlling inflammation, and enhancing the quality of life for many patients.

Despite these advances, accumulating evidence highlights the complex interplay and complementary roles of IL-17A and IL-17F in driving both acute and chronic phases of inflammatory disease. The differential expression patterns, cellular sources, and synergistic interactions with other inflammatory cytokines highlight the limitations of IL-17A monotherapy and provide a strong mechanistic basis for targeting both IL-17A and IL-17F.

Dual inhibition therapies have demonstrated enhanced clinical efficacy across various disease domains and appear to provide a more comprehensive approach to modulating IL–17–mediated inflammation. These findings suggest that dual IL-17A/F blockade may overcome the regulatory and pathogenic escape mechanisms inherent in single-cytokine targeting, potentially leading to improved and sustained patient outcomes. Ongoing and future head-to-head clinical trials and long-term safety and efficacy data will be essential to fully establish the role of dual IL-17 inhibition in treatment algorithms for PsA and SpA. Collectively, the evolution from single to dual IL-17 pathway targeting marks a promising advancement in precision medicine for inflammatory arthritides, offering the potential to significantly improve disease control and enhance patients’ quality of life.
